# Secondary malignancies and survival of FCR‐treated patients with chronic lymphocytic leukemia in Central Europe

**DOI:** 10.1002/cam4.5033

**Published:** 2022-10-07

**Authors:** Fruzsina Kósa, Tereza Nečasová, Martin Špaček, Krzysztof Giannopoulos, Iwona Hus, Tereza Jurková, Eva Koriťáková, Marika Chrápavá, Martina Nováčková, Ivana Katinová, Denisa Krejčí, Adam Jujka, Zoltán Mátrai, István Vályi‐Nagy, Tadeusz Robak, Michael Doubek

**Affiliations:** ^1^ Janssen Global Services LLC Budapest Hungary; ^2^ Institute of Biostatics and Analysis Ltd. Brno Czech Republic; ^3^ General University Hospital in Prague Prague Czech Republic; ^4^ Experimental Hematooncology Department Medical University of Lublin Lublin Poland; ^5^ Medical University of Lublin, Department of Clinical Transplantology Medical University of Lublin Lublin Poland; ^6^ Instutute of Hematology and Transfusion Medicine Warsaw Poland; ^7^ Institute of Biostatistics and Analyses of the Faculty of Medicine Brno Czech Republic; ^8^ Janssen‐Cilag Polska Warsaw Poland; ^9^ 1st Department of Internal Medicine – Haematology United St István and St László Hospital Budapest Hungary; ^10^ Department of Hematology Medical University of Lodz Lodz Poland; ^11^ University Hospital Brno and CEITEC Masaryk University Brno Czech Republic

**Keywords:** CLL population, FCR therapy, secondary malignancy, survival

## Abstract

This is the first large‐scale cross‐country analysis of patients with chronic lymphocytic leukemia (CLL) aimed to evaluate the incidence, types, and key prognostic factors of secondary malignancies, and to assess the impact on overall survival based on retrospective claims data from three Central European countries. We analyzed 25,814 newly diagnosed CLL patients from Czechia, Hungary, and Poland; 10,312 (39.9%) patients were treated for CLL in study periods between 2004 and 2016. Out of the treated patients, 1986 (19.3%) received the FCR therapy in the first line and 779 (7.6%) received FCR in subsequent lines. We observed that 33.7% of treated patients developed secondary malignancies during the study. Based on country estimates, the probability to develop a secondary malignancy within 4 years since starting the first‐line FCR therapy ranged between 28.0% and 36.8%. We found the age at diagnosis, male gender, any malignancy prior to the CLL diagnosis, and the CLL treatment to be the key risk factors for developing secondary malignancies. Specifically, the FCR therapy was a statistically significant (*p* < 0.001) prognostic factor for risk increase with the hazard ratio between 1.46 and 1.60. Across the three Central European countries, we observed consistent results indicating FCR increased the risk of secondary malignancies in CLL patients. We conclude that secondary malignancies are clearly an undervalued burden for CLL patients, caregivers, and the healthcare system. When evaluating new therapies in regulatory and reimbursement decision making, the factor of secondary malignancies deserves deeper considerations.

## Graphical abstract

We analyzed 25,814 newly diagnosed CLL patients from Czechia, Hungary, and Poland between 2004 and 2016. The probability to develop a secondary malignancy within 4 years from starting the first‐line FCR therapy ranged between 28.0% and 36.8% with age at diagnosis, male gender, any malignancy prior to the CLL diagnosis, and the CLL treatment to be the key risk factors. Specifically, the FCR therapy was a statistically significant (*p* < 0.001) prognostic factor for risk increase with the hazard ratio between 1.46 and 1.60 indicating that when evaluating new therapies in regulatory and reimbursement decision making, the factor of secondary malignancies deserves deeper considerations.

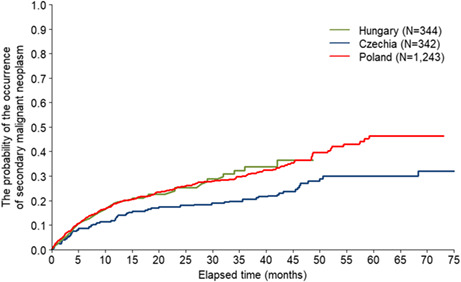

## 1 INTRODUCTION

Chronic lymphocytic leukemia (CLL) is the most common leukemia in the Western world. The incidence is followed up closely in the Central European countries, indicating an improvement from the situation a decade ago when the CzEch Leukemia Study Group – for Life (CELL) concluded the disease was often misdiagnosed and rather underreported.^1^

Since the year 2000, new medications have been studied to expand therapeutic options and to improve patient outcomes, for example, addition of rituximab to chemotherapy with fludarabine and cyclophosphamide (FCR),^2^ especially in patients with untreated CLL without TP53 abnormalities.^3^

There is exhaustive literature on standard outcomes of survival related to the disease and its treatments, however, the evidence of burden of secondary malignancies is still limited. CLL and related FCR treatment have been studied and found to increase the risk of developing secondary malignancies, predominantly melanoma and non‐melanoma skin cancers (NMSC),^4^, ^5^ acute myeloblastic leukemia (AML) and myelodysplastic syndromes (MDS),^6^ as well as other cancers, such as breast, prostate, lung, gastrointestinal, and head‐and‐neck tumors.^7^

Over the past years, the use of new therapies raised concerns of increased risks in treated CLL patients to develop secondary malignancies, namely, MDS, NMSC, AML, as well as other types, both due to treatment effect and a longer overall survival. A Canadian study reported an increased risk of secondary cancers in treated CLL patients compared with untreated ones stating the inherent predisposition to second cancers is further increased by treatment.^8^ Experts agree that, during the study period, FCR was the preferred first‐line treatment for CLL in Czechia accounting for a half of the cases, the other half of patients received rituximab with bendamustin or chlorambucil or corticoids. In Hungary and Poland, the first‐line treatments were chlorambucil, FCR, and R‐CHOP, in order.

In 2015, Benjamini et al. calculated the risk of secondary cancers in first‐line FCR‐treated CLL patients to be 2.38 times higher than in the general population, reducing overall survival in the FCR‐treated CLL patients.^9^ Moreover, the CLL therapy has been suspected to increase especially the risk of developing MDS^10^ and secondary AML.^10^, ^11^, ^12^ An increase in relative risk of NMSC has been reported in CLL patients with immunity dysfunction being the main cause.^13^ Secondary skin cancers typically develop in older age and are more aggressive in the CLL patients.^4^

While treatment extends the life of CLL patients, it results in a higher risk of complications and secondary malignancies. The literature evidence of the secondary malignancy incidence rate is limited, we have not found any registered active prospective study on long‐term complications of the CLL therapy.

This study is the first large‐scale cross‐country analysis of CLL patients to describe the incidence and types of secondary malignancies, to analyze key prognostic factors for developing them, and to assess the impact on overall survival. We analyzed differences across patient populations, healthcare environments, and treatment patterns in Central Europe.

We processed anonymized data only, hence, informed consents of the data subjects were waived. All ethical and regulatory approvals were obtained per applicable Hungarian, Czech, and Polish regulations.

## 2 METHODS

This was a retrospective non‐interventional claims database study conducted in parallel in Czechia, Hungary, and Poland based on methods harmonized across countries. Data on the diagnosis, FCR therapy (only, not other chemotherapies), and secondary malignancies were collected from the National Oncology Registry (NOR), payers' claim statements of two university hospitals, the General University Hospital in Prague and The University Hospital Brno, and the Deceased Examination Sheet in Czechia; from the National Health Insurance Fund (NEAK) database of demographics, claims, and the deceased in Hungary; and from the National Health Fund in Poland. A full population coverage was estimated in Poland (the total population about 37.8 million) and Hungary (the total population about 9.7 million). The population derived from the data of the two university hospitals in Czechia covered 59.8% incidence and 41.0% prevalence (mean over years) in the CLL patient population during the study follow‐up period.

### 2.1 Study objectives

The primary objective of the study was to describe epidemiology and survival in CLL patients with secondary malignancies with or without FCR therapy in Central Europe, with a special focus on NMSC, AML, and MDS. Our secondary objective was to identify key prognostic factors for developing secondary malignancies in CLL patients.

### 2.2 Inclusion criteria

Patients had to have at least two CLL diagnostic code (C9110) registrations (per the 10th revision of the International Statistical Classification of Diseases and Related Health Problems ‐ ICD‐10) during the study period at in‐ or outpatient care (in case of inpatient care, interventions were considered with the main ICD‐10 code only) and had to be at least 18 years old at the time of the first CLL ICD‐10 code registration.

### 2.3 Exclusion criteria

Patients had at least one CLL ICD‐10 code registered during 3 years prior to the eligibility assessment or had at least two mantle cell lymphoma (MCL) ICD code (C8200, C8310, C8300) registrations during the study period at in‐ or outpatient care (in case of inpatient care, interventions were considered with the main ICD codes only) one of which was prior to the first CLL ICD‐10 code registration.

Patients with diagnoses coded C82.0 (follicular lymphoma), C83.0 (small cell B‐cell lymphoma) or diagnosed after the first diagnosis of CLL were excluded. Richter's transformation was excluded by clinical presentation and histology examination in Czechia as well as based on diagnostic coding in Hungary and Poland.

### 2.4 Secondary malignancies of interest

Secondary malignancies of interest included MDS (code D46.X), NMSC (code C44.X), AML (code C92.0), and other malignancies (code CXX.X, where X represents any possible number) appearing with at least 2 ICD 10 code registrations during the study period at in‐ or outpatient care per study eligibility criteria.

### 2.5 Data collection

The follow‐up period was 2004–2013 in Hungary, 2008–2016 in Czechia, and 2010–2015 in Poland (Table S1).

We calculated the age at the time of the first registered CLL code during the follow‐up period and defined static age groups. Gender and the date of death were reported in all datasets.

As for treatments, the data collection protocol reflected differences in the routine clinical practice across countries. In Hungary, we included therapies with a CLL code registration, such as all chemotherapies, all drugs with L01 Anatomical Therapeutic Chemical Classification System (ATC) code, and special‐financed drugs (alemtuzumab, rituximab, bortezomib). In Czechia, we included the same drugs as in Hungary as well as therapies administered during the time when the CLL code was the dominant diagnosis registered in the hospital treatment records. In Poland, we included therapies with the C91.1, C91, and Z51.1 ICD code registration, that is, all chemotherapies, all drugs with the L01 ATC code, and special‐financed drugs.

FCR was available between 2009 and 2013 in Hungary and during the whole follow‐up period in Czechia and Poland.

**FIGURE 1:**
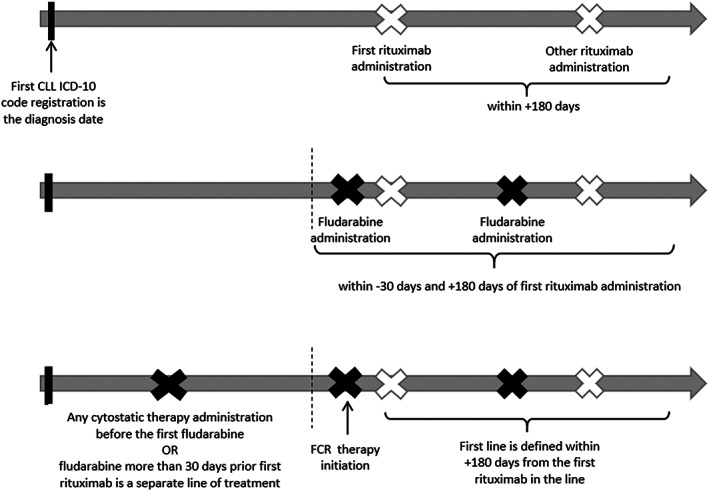
FCR therapy algorithm

We defined the FCR therapy algorithm as displayed in Figure 1. First, we identified the FCR therapy based on the following considerations. We searched for two rituximab administrations in the patient's pathway (as normal drug dispensation or special‐financed drug code) within 180 days, then we searched for two fludarabine therapy codes (at inpatient care or as drug dispensation code) within 30 days before and 180 days after the first rituximab administration. In the second step, we categorized the first‐line or a later‐line FCR. If there was any other therapy (listed in “Therapies worksheet”) or fludarabine treatment within more than 30 days prior to the first rituximab administration, then FCR was categorized as at least the second line, otherwise it was considered as first‐line FCR treatment. All FCR therapy components had to be registered with the CLL 9110 code in Hungary and Czechia, and with C91.1, C91, or Z51.1 ICD codes in Poland.

The types of secondary malignancies included in the study were independent of each other, one patient could have more than one type registered during the follow‐up period. We examined the first occurrences only, malignancies that occurred in the patient's history before the follow‐up period were excluded from analysis. To measure the level of association between the variables, we calculated their absolute and relative frequencies for each reasonable comparison. For statistical analyses comparing a single categorical variable among countries, we performed the Fisher's test.

### 2.6 Kaplan–Meier curves

We assessed the time from the first‐line FCR therapy initiation after the first CLL diagnosis to the first secondary malignant neoplasm or to death with time‐to‐event analysis plotting Kaplan–Meier curves. For mortality, the start‐point was the first‐line FCR therapy initiation after the first CLL diagnosis and the stop‐time was death, we censored data at the end of the follow‐up period.

### 2.7 Cox model

We analyzed the effect of the covariates on the hazard of developing the first secondary malignancy by the Cox proportional hazards model. All patients were observed from the diagnosis to the first secondary malignancy, death, or the end of the follow‐up period. The significance level was 5.0%.

Covariates in the Cox model were gender, the age at diagnosis, a previous malignant neoplasm prior to the CLL diagnosis, and static treatment status groups (no treatment, treated without FCR, treated with FCR). The *p*‐values were added to show the significance of results.

### 2.8 Missing data

We excluded missing data from the relevant analyses and did not utilize any data imputation techniques.

## 3 RESULTS

### 3.1 Total CLL population

In the total study population of 25,814 newly diagnosed CLL patients, 10,312 (39.9%) patients were treated for CLL. Out of the treated patients, 1986 (19.3%) received FCR in the first line and 779 (7.6%) received FCR in subsequent lines. Baseline characteristics are summarized in Table 1. While almost all Czech patients received their FCR therapy in the first line, Hungarian and Polish patients receive it equally in the first line and in subsequent lines. We analyzed separately patients with the first‐line FCR therapy to account for this difference in treatment patterns across countries. The number of patients diagnosed annually in each country was stable and did not change significantly over time. The annual mortality in the total CLL population varied between 5.9% and 9.3%. We did not find any clinically significant differences in age/gender demographics of the Hungarian and Polish patients at the diagnosis or at the start of therapy. Czech patients were younger than the Hungarian and Polish ones both at diagnosis and at their treatment start, and more male patients were diagnosed (Table S2). The Polish patients were generally younger than patients in Czechia and Hungary when starting the first‐line FCR therapy. The characteristics of deceased patients is presented in the Table S3. We did not observe any other statistically significant cross‐country differences in demographics.

**TABLE 1:** Baseline characteristics of total population

	All CLL patients			Treated patients			First‐line FCR‐treated patients		
	HU	CZ	PL	HU	CZ	PL	HU	CZ	PL
Number of patients (*N* [% of total/ treated patients])	8442 (100)	3574 (100)	13,798 (100)	2916 (34.5)	941 (26.3)	6455 (46.8)	344 (4.1/11.8)	399 (11.2/42.4)	1243 (9.0/19.3)
Male (*N* [%])	4628 (54.8)	2199 (61.5)	7781 (56.4)	1722 (59.1)	596 (63.3)	3943 (61.1)	229 (66.6)	270 (67.7)	845 (68.0)
Age groups (*N* [%])									
18–29	60 (0.7)	44 (1.2)	95 (0.7)	13 (0.4)	2 (0.2)	41 (0.6)	< 10 (<2.9)	0 (0.0)	6 (0.5)
30–39	126 (1.5)	67 (1.9)	143 (1.0)	37 (1.3)	9 (1.0)	60 (0.9)	< 10 (<2.9)	4 (1.0)	23 (1.9)
40–49	438 (5.2)	165 (4.6)	609 (4.4)	179 (6.1)	43 (4.6)	327 (5.1)	40 (11.6)	25 (6.3)	140 (11.3)
50–59	1506 (17.8)	606 (17.0)	2339 (17.0)	610 (20.9)	178 (18.9)	1247 (19.3)	113 (32.8)	114 (28.6)	466 (37.5)
60–69	2537 (30.1)	1275 (35.7)	4427 (32.1)	948 (32.5)	383 (40.7)	2080 (32.2)	121 (35.2)	187 (46.9)	480 (38.6)
70–79	2652 (31.4)	1075 (30.1)	4092 (29.7)	864 (29.6)	284 (30.2)	1848 (28.6)	54 (15.7)	65 (16.3)	122 (9.8)
80–89	1059 (12.5)	325 (9.1)	1967 (14.3)	253 (8.7)	42 (4.5)	803 (12.4)	11 (3.2)	4 (1.0)	6 (0.5)
90+	64 (0.8)	17 (0.5)	126 (0.9)	12 (0.4)	0 (0.0)	49 (0.8)	< 10 (<2.9)	0 (0.0)	0 (0.0)
Deaths during follow‐up (*N* [%])	3302 (39.1)	1147 (32.1)	3112 (22.6)	1489 (51.1)	338 (35.9)	2033 (31.5)	44 (12.8)	122 (30.6)	187 (15.0)

### 3.2 Secondary malignancies

We noticed 33.7% of treated CLL patients developed secondary malignancies in the three countries. In Czechia, fewer patients (22.9%) were diagnosed with any secondary malignancy in the total CLL population, and the differences in the rates of secondary neoplasms in the treated population were statistically significant across the countries (Table 2). In average, we observed 1.24 independent secondary malignancies per patient with the highest probability of incidence in the first relative year after the CLL diagnosis. Average cross‐country incidence rates were 2.3% and 1.7% for AML, 1.2% and 1.1% for MDS, and 3.4% and 3.6% for NMSC in the total CLL and treated CLL populations, respectively.

**TABLE 2:** Secondary malignancies in all CLL patients

	Total CLL population					Treated population				
	Total CLL population	Patients with any secondary malignancy	Patients with AML	Patients with MDS	Patients with NMSC	Treated population	Patients with any secondary malignancy	Patients with AML	Patients with MDS	Patients with NMSC
HU (*N* [%])	8442 (100.0)	3299 (39.1)	182 (2.2)	109 (1.3)	480 (5.7)	2916 (100.0)	1091 (37.4)	47 (1.6)	28 (1.0)	163 (5.6)
CZ (*N* [%])	3574 (100.0)	818 (22.9)	145 (4.1)	63 (1.8)	44 (1.2)	941 (100.0)	326 (34.6)	27 (2.9)	17 (1.8)	20 (2.1)
PL (*N* [%])	13,798 (100.0)	4568 (33.1)	102 (0.7)	94 (0.7)	457 (3.3)	6455 (100.0)	2056 (31.9)	38 (0.6)	27 (0.4)	197 (3.1)
Total (*N* [%])	25,814 (100.0)	8685 (33.6)	429 (1.7)	266 (1.0)	981 (3.8)	10,312 (100.0)	3473 (33.7)	112 (1.1)	72 (0.7)	380 (3.7)
*p*‐value		<0.001	<0.001	<0.001	<0.001		<0.001	<0.001	<0.001	<0.001

*Note*: The *p*‐values for Fisher's tests are linked to the number of patients in categories in Czechia, Hungary, and Poland.

Cross‐country demographic differences at the time of the secondary malignancy diagnosis are presented in the Table S4. In the total CLL population, we noticed the highest rate of patients with secondary malignancies in the age group 60–69 years (36.2%) in Czechia, and in the age group 70–79 years in Hungary (41.9%) and Poland (44.5%). In the treated population, we saw the highest rate of secondary malignancies in the age group 60–69 years in Czechia (41.7%), and in patients older than 70 years (39.3%) in Poland. We observed the highest rate of patients with secondary malignancies in the first‐line FCR‐treated population in Polish patients younger than 60 years (45.0%), while in Czechia and Hungary it was in the age group 60–69 years (52.3% and 45.7%, respectively).

While AML was typically diagnosed at a younger age below 60 years, MDS and NMSC were mostly diagnosed in the population older than 70 years (Table S5).

The mortality in CLL patients with secondary malignancies was highest in the age group 70–79 years in both Hungary (42.0%) and Poland (35.3%), while in Czechia most patients died in the age group 60–69 years (36.2%) (Table S6).

In the total CLL population, the most frequent types of secondary malignancies were leukemia (11.0%), non‐Hodgkin lymphoma (10.8%), and NMSC (3.8%); the order and magnitude of prevalence in the treated population showed a similar pattern (Table 3).

**TABLE 3:** Raw frequency of secondary malignancies before modeling for multiple predictors and different follow‐up times

	Total CLL population				Treated population				First‐line FCR‐treated population			
	HU *N* = 8442	CZ *N* = 3574	PL *N* = 13,798	Total *N* = 25,814	HU *N* = 2916	CZ *N* = 941	PL *N* = 6455	Total *N* = 10,312	HU *N* = 344	CZ *N* = 399	PL *N* = 1243	Total *N* = 1986
Leukemia (*N* [%])	700 (8.3)	289 (8.1)	1848 (13.4)	2837 (11.0)	221 (7.6)	101 (10.7)	798 (12.4)	1120 (10.9)	10 (2.9)	43 (10.8)	111 (8.9)	164 (8.3)
Non‐Hodgkin lymphoma (*N* [%])	860 (10.2)	269 (7.5)	1664 (12.1)	2793 (10.8)	208 (7.1)	122 (13.0)	787 (12.2)	1117 (10.8)		48 (12.0)	114 (9.2)	162 (8.2)
Other malignant neoplasms of skin (NMSC) (*N* [%])	480 (3.8)	44 (1.2)	457 (3.3)	981 (3.8)	163 (4.3)	20 (2.1)	197 (3.1)	380 (3.7)			24 (1.9)	24 (1.2)
Multiple myeloma (*N* [%])	178 (2.1)	98 (2.7)	298 (2.2)	574 (2.2)	92 (3.2)	51 (5.4)	121 (1.9)	264 (2.6)			17 (1.4)	17 (0.9)
Malignant neoplasm of colon, rectosigmoid and rectum (*N* [%])	140 (1.7)	32 (0.9)	228 (1.7)	400 (1.5)	29 (1.0)		91 (1.4)	120 (1.2)				
Malignant neoplasm of prostate (*N* [%])	69 (0.8)	25 (0.7)	267 (1.9)	361 (1.4)			96 (1.5)	96 (0.9)			14 (1.1)	14 (0.7)
Hodgkin lymphoma (*N* [%])	76 (0.9)	60 (1.7)	216 (1.6)	352 (1.4)	37 (1.3)	33 (3.5)	99 (1.5)	169 (1.6)			13 (1.0)	13 (0.7)
Malignant neoplasm of trachea, bronchus, and lung (*N* [%])	119 (1.4)	34 (1.0)	193 (1.4)	346 (1.3)	36 (1.2)	13 (1.4)	99 (1.5)	148 (1.4)			12 (1.0)	12 (0.6)
Malignant neoplasm of breast (*N* [%])	71 (0.8)	15 (0.4)	165 (1.2)	251 (1.0)	19 (0.7)		52 (0.8)	71 (0.7)				
Malignant neoplasm of kidneys [*N* (%)]	22 (0.3)	17 (0.5)	119 (0.9)	158 (0.6)			41 (0.6)	41 (0.4)				
Malignant melanoma of skin (*N* [%])	38 (0.5)	16 (0.4)	69 (0.5)	123 (0.5)			32 (0.5)	32 (0.3)				
Malignant neoplasm of lip and pharynx (*N* [%])	45 (0.5)	13 (0.4)	10 (0.1)	68 (0.3)	15 (0.5)			15 (0.1)				
Malignant neoplasm of brain, spinal cord, and other parts of CNS (*N* [%])	18 (0.2)	11 (0.3)	38 (0.3)	67 (0.3)			13 (0.2)	13 (0.1)				
Benign neoplasm and neoplasms of uncertain or unknown behavior (*N* [%])		63 (1.8)		63 (0.2)		17 (1.8)		17 (0.2)				
Other malignant neoplasm (*N* [%])	529 (6.3)	64 (1.8)	819 (5.9)	1412 (5.5)	185 (6.3)	18 (1.9)	278 (4.3)	481 (4.7)	11 (3.2)			11 (0.6)

### 3.3 Time‐to‐event analysis

**FIGURE 2:**
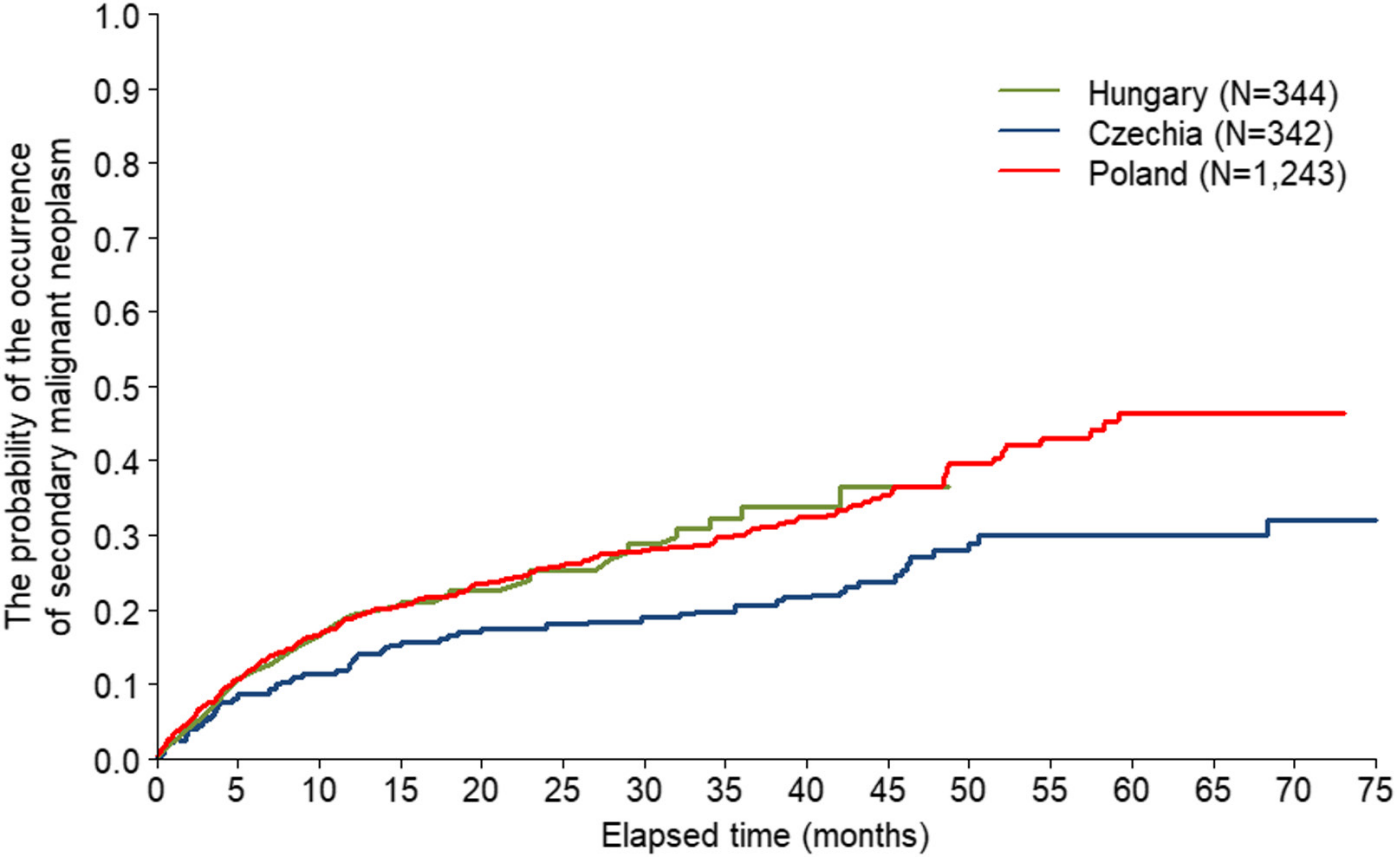
Kaplan–Meier curve to the first secondary malignant neoplasm after the first‐line FCR therapy initiation post‐CLL diagnosis

**FIGURE 3:**
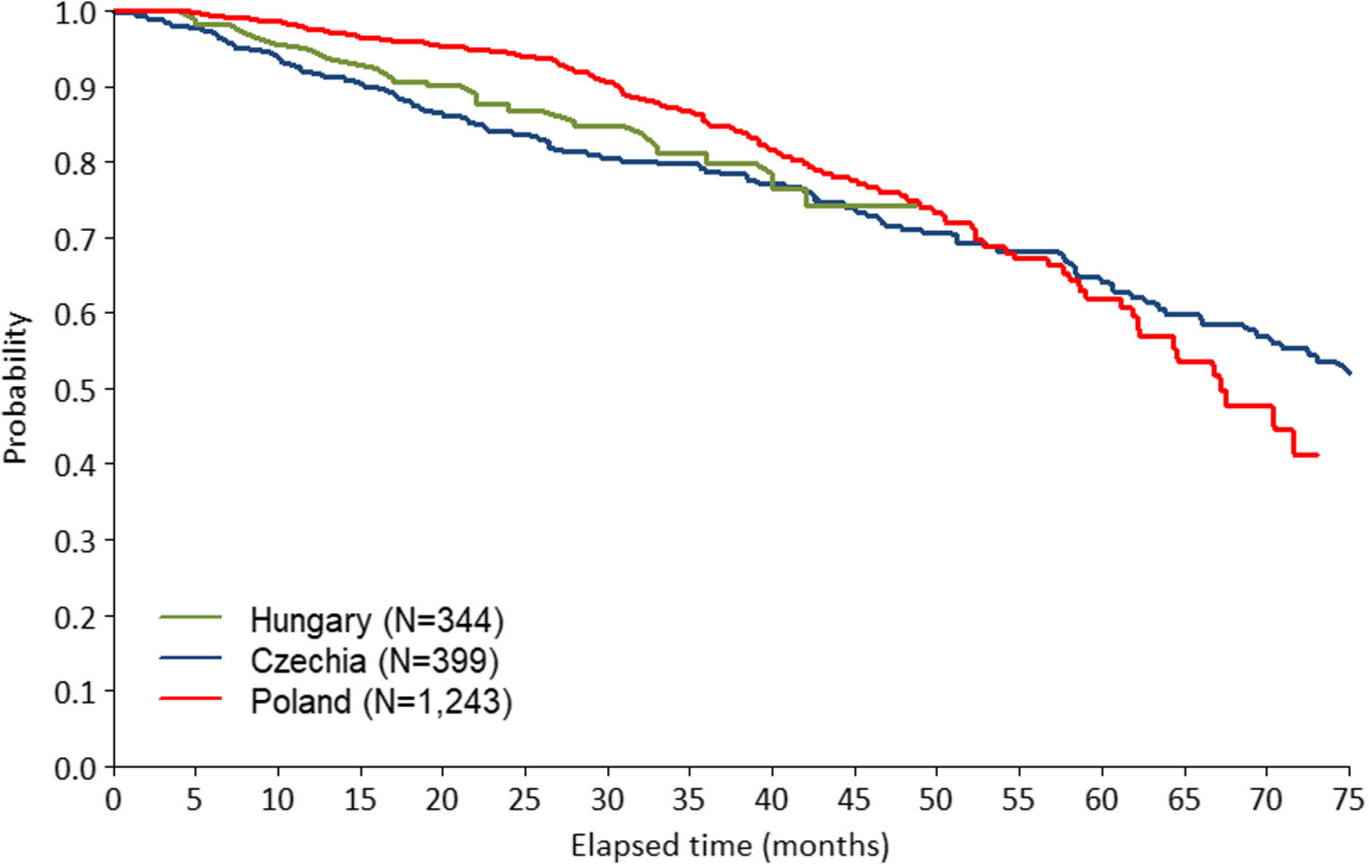
Mortality in the first‐line FCR therapy population

The Kaplan–Meier curve in Figure 2 shows the time from the start of the first‐line FCR therapy to the first occurrence of a secondary malignant neoplasm. The mortality in the first‐line FCR‐treated population is displayed in Figure 3. Based on country estimates, the probability to develop any secondary malignancy within 4 years since starting the first‐line FCR therapy ranged between 28.0% and 36.8%. The 5‐year estimate ranged between 29.9% and 36.4% in Czechia and Poland.

### 3.4 Risk factors

The results from the cross‐country Cox models are summarized in Table 4. We found the age at diagnosis, male gender, a previous malignancy prior to the CLL diagnosis, and CLL treatment were the key prognostic risk factors for developing secondary malignancies after the CLL diagnosis. The FCR treatment was a statistically significant (*p* < 0.001) covariate of an increased risk to develop secondary malignancies in all three countries with a hazard ratio varying between 1.46 and 1.60 (Figure S1).

**TABLE 4:** Risk factors to develop any secondary malignancy development, Cox model of selected variables in all countries

	HU		CZ		PL	
Covariates	HR	*p*‐value	HR	*p*‐value	HR	*p*‐value
Age						
18–49	0.93	0.378	2.14	**<0.001**	1.31	**<0.001**
50–59	1.00	—	1.00	—	1.00	—
60–69	1.04	0.429	0.96	0.66	1.05	0.275
70+	1.05	0.353	0.83	0.08	1.06	0.175
Gender						
Male	1.00	—	1.00	—	1.00	—
Female	0.83	**<0.001**	0.98	0.76	0.94	0.031
No previous malignant neoplasm	1.00	—			1.00	—
Previous malignant neoplasm	1.38	**<0.001**			3.28	**<0.001**
Not treated for CLL	1.00	—	1.00	—	1.00	—
Therapy, no FCR	1.76	**<0.001**	2.13	**<0.001**	1.22	**<0.001**
FCR therapy	1.46	**<0.001**	1.57	**<0.001**	1.59	**<0.001**

Bold values presented in the original analysis, it is not possible to re‐run the analysis any more.

## 4 DISCUSSION

In our sample of data of 25,814 patients from Czechia, Hungary, and Poland collected between 2004 and 2016, about one third of the CLL patients treated with any CLL therapy developed a secondary malignancy in the total follow‐up period. We found the age, gender, a previous malignancy prior to the CLL diagnosis, and any CLL treatment to be the most important risk factors for developing secondary malignancy in the CLL population.

We found Hungary and Poland comparable in terms of patient demographics and treatment patterns driven by daily clinical practice. In Czechia, patients were diagnosed at a younger age than in the other countries and the ratio of untreated patients in the total sample was higher, the FCR therapy was more common, mostly in the first line. The differences in treatment patterns can be explained by the fact the access to therapy is easier in Czechia, e.g., FCR is also available in outpatient care, as well as by considering the completeness of data, namely, covering the total population in Hungary and Poland, while in Czechia the data were limited to the two major university hospitals in Prague and Brno with an estimated 60% coverage of the national CLL incidence in the population with a bias toward more severe cases.

In patients treated with any therapy, we observed a 32.3% chance to develop secondary malignancies within 4 years from the therapy start. This is in line with results published by other researchers who reported a 1.7‐fold increased risk of second cancers compared with untreated CLL patients,^8^ and the cumulative frequency of other cancers about 36% in long‐term survivors of CLL irrespective of treatment.^7^ Two recent studies put the highest risk of secondary malignancies within 2–12 months after the CLL diagnosis,^14^, ^15^ which corresponds to our finding of the highest incidence of secondary malignant neoplasms reported mostly in the first relative year. Although we assume a closer follow‐up in the first year after treatment start, no major bias is expected from population‐level national claims data where malignancies are reported in routine clinical practice.

While the most prevalent cancers in general population are lung, breast, colorectal, prostate, non‐melanoma skin, and stomach cancers,^16^ we observed leukemia (defined by ICD‐10 codes of C91‐C95), non‐Hodgkin lymphoma, non‐melanoma skin cancer, multiple myeloma, and colorectal cancer as the most prevalent secondary malignancies in the CLL population both in the treated and untreated patients, which confirms the expert opinion that the CLL diagnosis as well as therapy make patients prone to develop specific cancer types. We found secondary AML diagnosed mostly in younger patients below 60 years, and secondary MDS and NMSC diagnosed mostly in the fragile ones above 70 years of age.

We should consider that CLL patients do not have secondary malignancies only but other primary malignancies as well. Treatment prolongs patients' survival, hence, there is more time for other primary malignancies to develop. We cannot exclude the possibility that CLL patients may be predisposed to develop malignancies although a clear genetic evidence has not been found yet. We faced challenges to identify true second primary malignancies from the claims data. The data dictionary of comorbid ICD10 codes was aligned across the three countries to mirror the daily clinical practices and to avoid counting miscoded CLL as second primary malignancy. To the best of our knowledge, the final data dictionary contained the smallest bias for the cross‐country comparison. However, we are sure there was a bias in miscoding CLL with other malignancies, therefore, we present the full table of malignancies to be transparent.

The probability of overall survival in CLL patients was comparable across countries for all treatment modalities, the median survival was not reached in any of the follow‐up periods.

An important future direction would be to refine the present analysis with a focus on the FCR‐treated population, especially from data sources where explicit information is available on the treatment lines.

### 4.1 Limitations

Although all three Central European countries have similar populations in terms of genetics and healthcare systems, the comparison of data was difficult due to different coding systems and prescribing models for in‐ and outpatient care as well as the different follow‐up length in each country. The claims data we analyzed had no qualitative information, such as severity classification, diagnostic test results, or cause of death. To comply with the Hungarian National Health Insurance Fund's data protection policy, we could not obtain exact data if the number of patients was less than 10; in these cases, statistical testing was not performed. In Czechia, most patients were included in the study, as they are treated in the two hospitals providing the data. We could not calculate confidence intervals since primary data were not available. Data on previous malignancies were not available in Czechia. AMS, NMSC, and MDS patients were not analyzed deeper or modeled for risk factors for secondary malignancies due to low sample sizes in all countries.

Among the descriptive statistics of the population the median length of follow‐up was not reported in the original country‐level analyses where revisiting the data was not possible.

Causality derived from the Cox models between the survival and the treatment are limited as there were possibly unmeasured confounders that we did not account for due to the nature of claims data.

## 5 CONCLUSIONS

This is the first large‐scale analysis of its kind with a harmonized cross‐country approach to payer data. Across the three Central European countries, we observed consistent results indicating that treatment increased the risk of secondary malignancies in CLL patients. The FCR therapy, a preferred choice of clinicians in both the first and subsequent lines of treatment in the region during the study period, carried an increased risk to develop secondary malignancies. Clinicians should carefully consider these risks while making decisions on the therapeutic approach and treatment modalities in their patients.

We conclude that secondary malignancies are clearly an undervalued burden for CLL patients, caregivers, and the healthcare system. When evaluating new therapies in regulatory and reimbursement decision making, the factor of secondary malignancies deserves deeper considerations.

## AUTHOR CONTRIBUTIONS

FK was involved in the concept development from the first study in Hungary and had been coordinating the overall project. She reviewed the research plan and supported it with literature review. She worked on the acquisition of data in Hungary and supported the drafting of the publication plan. She analyzed the aggregate results, drafted the discussion and critically reviewed and submitted the final paper. AJ worked on the data acquisition and analysis for Poland, he reviewed the joint protocol, the results from Poland and the joint results, he supported manuscript writing with country‐specific input, he worked on the publication plan and he reviewed and approved the final manuscript. TN, TJ, EK, IK, and DK have been the statistical team responsible for data preparation in Poland and Czechia, for developing the statistical plan, for analysis of data from Poland, Czechia and for providing statistical background for the joint report. They reviewed and approved the final manuscript.

MN have been responsible for project and research plan coordination in Czechia and Poland, for data acquisition in Czechia, for results merger, critical review of the results and the manuscript and final approval of the publication. MC worked on the concept development, the protocol and research plan development for the analysis in Poland and Czechia and the joint analysis, reviewed the manuscript drafts and approved the final publication. MZ and MS supported the statistical plan. MZ, MS, IH, and KG supported the protocol planning and the background and discussion sections in the manuscript with literature references. IVN, TR, and MD had supported the cross‐country data merger process. MZ and IVN in Hungary, KG, IH, and TR in Poland and MS and MD in Czechia were responsible for the clinical support for the studies in their respective countries of operation and were part of the concept development team for the cross‐country study. They reviewed the statistical plan, the study protocol, the cross‐country report with the draft publication plan and commented and critically reviewed the final manuscript.

## FUNDING INFORMATION

This study was funded by Janssen. The work of the authors István Vályi‐Nagy and Zoltán Mátrai were also supported by the grant NVKP_16–2016‐0005 by the Hungarian National Agency for Research, Development and Innovation.

## CONFLICT OF INTEREST

Michael Doubek: Honoraria and research grants: AbbVie, Amgen, AOP Orphan, Gilead, Janssen‐Cilag, Novartis, Pfizer, Roche. Martin Špaček: Honoraria: AbbVie, Gilead, Janssen‐Cilag, Roche. Martina Nováčková, Marika Chrápavá, Tereza Nečasová, Tereza Jurková, Eva Koriťáková, Ivana Katinová, Denisa Krejčí are employees of the Institute of Biostatistics and Analyses Ltd. that received funding from Janssen for the participation in the study. Fruzsina Kósa and Adam Jujka are employees of Janssen.

## ETHICAL STATEMENT

The manuscript reflects the authors' own opinion. It is an original research and analysis which has not been previously published elsewhere.

## Supporting information


Table S1

Table S2

Table S3

Table S4

Table S5

Table S6

Figure S1

## Data Availability

This is a retrospective secondary database study, no new data were generated or analyzed in support of this research.
