# Supplementary material for: Secondary malignancies and survival of FCR‐treated patients with chronic lymphocytic leukemia in Central Europe
Source: Cancer Med. 2022 Oct 7;12(2):1961–71. doi: 10.1002/cam4.5033 (PMC9883578; doi:10.1002/cam4.5033)
Supplement: Supplementary file 1 — Table S1 [file CAM4-12-1961-s002.doc]

Supplementary Table 1. Study and follow-up period per country

| **Country** | **Study period** | **Diagnosis-free period** | **Follow-up period** |
| --- | --- | --- | --- |
| Hungary | 01 Jan 2000 – 31 Dec 2013 | 01 Jan 2000 – 31 Dec 2003 | 01 Jan 2004 – 31 Dec 2013 |
| Czechia | 01 Jan 2007 – 31 Dec 2016 | 01 Jan 2007 – 31 Dec 2007 | 01 Jan 2008 – 31 Dec 2016 |
| Poland | 01 Jan 2008 – 31 Dec 2015 | 01 Jan 2008 – 31 Dec 2009 | 01 Jan 2010 – 31 Dec 2015 |
