# Supplementary material for: Secondary malignancies and survival of FCR‐treated patients with chronic lymphocytic leukemia in Central Europe
Source: Cancer Med. 2022 Oct 7;12(2):1961–71. doi: 10.1002/cam4.5033 (PMC9883578; doi:10.1002/cam4.5033)
Supplement: Supplementary file 2 — Table S2 [file CAM4-12-1961-s005.docx]

Supplementary Table 2. Cross-country demographic differences at baseline

|  | **Total CLL population at diagnosis** | | | | | **Treated population at treatment start** | | | | | **First-line FCR treated population at First-line treatment start** | | | | |
| --- | --- | --- | --- | --- | --- | --- | --- | --- | --- | --- | --- | --- | --- | --- | --- |
|  | **Age** | | | **Gender** | **Total** | **Age** | | | **Gender** | **Total** | **Age** | | | **Gender** | **Total** |
|  | **<60** | **60─69** | **≥70** | **Male** |  | **<60** | **60─69** | **≥70** | **Male** |  | **<60** | **60─69** | **≥70** | **Male** |  |
| **HU [N (%)]** | 2,130 (25.2) | 2,537 (30.1) | 3,775 (44.7) | 4,628 (54.8) | 8,442 (100.0) | 839  (28.8) | 948  (32.5) | 1,129 (38.7) | 1,722 (59.1) | 2,916 (100.0) | 157  (45.6) | 121  (35.2) | 66  (19.2) | 229  (66.6) | 344 (100.0) |
| **CZ [N (%)]** | 882 (24.7) | 1,275 (35.7) | 1,417 (39.6) | 2,199 (61.5) | 3,574 (100.0) | 232  (24.7) | 383  (40.7) | 326  (34.6) | 596  (63.3) | 941 (100.0) | 143 (35.8) | 187  (46.9) | 69  (17.3) | 270  (67.7) | 399 (100.0) |
| **PL [N (%)]** | 3,186 (23.1) | 4,427 (32.1) | 6,185 (44.8) | 7,781 (56.4) | 13,798 (100.0) | 1,675 (25.9) | 2,080 (32.2) | 2,700 (41.8) | 3,943 (61.1) | 6,455 (100.0) | 635  (51.1) | 480  (38.6) | 128  (10.3) | 845  (68.0) | 1,243 (100.0) |
| **Total [N (%)]** | 6,198 (24.0) | 8,239 (31.9) | 11,377 (44.1) | 14,608 (56.6) | 25,814 (100.0) | 2,746 (26.6) | 3,411 (33.1) | 4,155 (40.3) | 6,261 (60.7) | 10,312 (100.0) | 935 (47.1) | 788 (39.7) | 263 (13.2) | 1,344 (67.7) | 1,986 (100.0) |
| **p-value** | < 0.001 | | | < 0.001 |  | < 0.001 | | | 0.040 |  | < 0.001 | | | 0.877 |  |

*The p-values for Fisher's tests are linked to the number of patients in categories in Czechia, Hungary, and Poland.*
