# Supplementary material for: Secondary malignancies and survival of FCR‐treated patients with chronic lymphocytic leukemia in Central Europe
Source: Cancer Med. 2022 Oct 7;12(2):1961–71. doi: 10.1002/cam4.5033 (PMC9883578; doi:10.1002/cam4.5033)
Supplement: Supplementary file 3 — Table S3 [file CAM4-12-1961-s007.docx]

Supplementary Table 3. Cross-country demographic differences at death

|  | **Total CLL population  at death** | | | | | **Treated population  at death** | | | | | **First-line FCR treated population  at death** | | | | |
| --- | --- | --- | --- | --- | --- | --- | --- | --- | --- | --- | --- | --- | --- | --- | --- |
|  | **Age** | | | **Gender** | **Total** | **Age** | | | **Gender** | **Total** | **Age** | | | **Gender** | **Total** |
|  | **<60** | **60─69** | **≥70** | **Male** |  | **<60** | **60─69** | **≥70** | **Male** |  | **<60** | **60─69** | **≥70** | **Male** |  |
| **HU [N (%)]** | 479 (14.5) | 831  (25.2) | 1,992 (60.3) | 1,940 (58.8) | 3,302  (100.0) | 291  (19.5) | 443  (29.8) | 755  (50.7) | 883  (59.3) | 1,489 (100.0) | - | 10  (22.7) | - | 30  (68.2) | 44 (100.0) |
| **CZ [N (%)]** | 117 (10.2) | 292  (25.5) | 738  (64.3) | 757  (66.0) | 1,147 (100.0) | 31  (9.2) | 107  (31.7) | 200  (59.2) | 224  (66.3) | 338 (100.0) | 18  (14.8) | 45  (36.9) | 59  (48.4) | 91  (74.6) | 122 (100.0) |
| **PL [N (%)]** | 419 (13.5) | 780  (25.1) | 1,913 (61.5) | 1,960 (63.0) | 3,112 (100.0) | 345  (17.0) | 559  (27.5) | 1,129 (55.5) | 1,295  (63.7) | 2,033 (100.0) | 71  (38.0) | 84  (44.9) | 32  (17.1) | 129  (69.0) | 187 (100.0) |
| **Total [N (%)]** | 1,015 (13.4) | 1,903 (25.2) | 4,643 (61.4) | 4,657 (61.6) | 7,561 (100.0) | 667 (17.3) | 1,109 (28.7) | 2,084 (54.0) | 2,402 (62.2) | 3,860 (100.0) | - | 139 (39.4) | - | 250 (70.8) | 353 (100.0) |
| **p-value** | 0.004 | | | < 0.001 |  | < 0.001 | | | 0.008 |  | - | | | 0.525 |  |
| In Hungary, the numbers could not be obtained due to privacy regulations if less than 10 patients were reported. | | | | | | | | | | | | | | | |

*The p-values for Fisher's tests are linked to the number of patients in categories in Czechia, Hungary, and Poland.*
