# Supplementary material for: Secondary malignancies and survival of FCR‐treated patients with chronic lymphocytic leukemia in Central Europe
Source: Cancer Med. 2022 Oct 7;12(2):1961–71. doi: 10.1002/cam4.5033 (PMC9883578; doi:10.1002/cam4.5033)
Supplement: Supplementary file 4 — Table S4 [file CAM4-12-1961-s001.docx]

Supplementary Table 4. Cross-country demographic differences at the time of secondary malignancy diagnosis

|  | **Total CLL population with secondary malignancy** | | | | | **Treated population with secondary malignancy** | | | | | **First-line FCR-treated population with secondary malignancy** | | | | |
| --- | --- | --- | --- | --- | --- | --- | --- | --- | --- | --- | --- | --- | --- | --- | --- |
|  | **Age** | | | **Gender** | **Total** | **Age** | | | **Gender** | **Total** | **Age** | | | **Gender** | **Total** |
|  | **<60** | **60─69** | **≥70** | **Male** |  | **<60** | **60─69** | **≥70** | **Male** |  | **<60** | **60─69** | **≥70** | **Male** |  |
| **HU [N (%)]** | 882 (26.7) | 1,034 (31.3) | 1,383 (41.9) | 1,902  (57.7) | 3,299 (100.0) | - | 358  (32.8) | - | 661  (60.6) | 1,091 (100.0) | - | 37  (45.7) | - | 53  (65.4) | 81 (100.0) |
| **CZ [N (%)]** | 263 (32.2) | 296  (36.2) | 259  (31.7) | 509  (62.2) | 818 (100.0) | 87  (26.7) | 136  (41.7) | 103  (31.6) | 215  (66.0) | 326 (100.0) | 43  (33.1) | 68  (52.3) | 19  (14.6) | 88  (67.7) | 130 (100.0) |
| **PL [N (%)]** | 1,104 (24.2) | 1,433 (31.4) | 2,031  (44.5) | 2,695 (59.0) | 4,568 (100.0) | 564  (27.4) | 684  (33.3) | 808  (39.3) | 1,302 (63.3) | 2,056 (100.0) | 145  (45.0) | 135  (41.9) | 42  (13.0) | 222  (68.9) | 322 (100.0) |
| **Total [N (%)]** | 2,249 (25.9) | 2,763 (31.8) | 3,673 (42.3) | 5,106 (58.8) | 8,685 (100.0) | - | 1,178 (33.9) | - | 2,178 (62.7) | 3,473 (100.0) | - | 240 (45.0) | - | 363 (68.1) | 533 (100.0) |
| **p-value** | < 0.001 | | | 0.054 |  | - | | | 0.144 |  | - | | | 0.810 |  |
| In Hungary, the numbers could not be obtained due to privacy regulations if less than 10 patients were reported. | | | | | | | | | | | | | | | |

*The p-values for Fisher's tests are linked to the number of patients in categories in Czechia, Hungary, and Poland.*
