# Supplementary material for: Secondary malignancies and survival of FCR‐treated patients with chronic lymphocytic leukemia in Central Europe
Source: Cancer Med. 2022 Oct 7;12(2):1961–71. doi: 10.1002/cam4.5033 (PMC9883578; doi:10.1002/cam4.5033)
Supplement: Supplementary file 5 — Table S5 [file CAM4-12-1961-s006.docx]

Supplementary Table 5. Cross-country demographic differences in patients with AML, MDS, or NMSC

|  | **Patients with AML** | | | | | **Patients with MDS** | | | | | **Patients with NMSC** | | | | |
| --- | --- | --- | --- | --- | --- | --- | --- | --- | --- | --- | --- | --- | --- | --- | --- |
|  | **Age at AML diagnosis** | | | **Gender** | **Total** | **Age at AML diagnosis** | | | **Gender** | **Total** | **Age at AML diagnosis** | | | **Gender** | **Total** |
|  | **<60** | **60─69** | **≥70** | **Male** |  | **<60** | **60─69** | **≥70** | **Male** |  | **<60** | **60─69** | **≥70** | **Male** |  |
| **HU [N (%)]** | 85 (46.7) | 42 (23.1) | 55 (30.2) | 111 (61.0) | 182 (100.0) | 19 (17.4) | 32 (29.4) | 58 (53.2) | 52 (47.7) | 109 (100.0) | 56 (11.7) | 166 (34.6) | 258 (53.8) | 291 (60.6) | 480 (100.0) |
| **CZ [N (%)]** | 84 (57.9) | 42 (29.0) | 19 (13.1) | 84 (57.9) | 145 (100.0) | 16 (25.4) | 19 (30.2) | 28 (44.4) | 38 (60.3) | 63 (100.0) | 2  (4.5) | 14 (31.8) | 28 (63.6) | 32 (72.7) | 44 (100.0) |
| **PL [N (%)]** | 42 (41.2) | 28 (27.5) | 32 (31.4) | 60 (58.8) | 102 (100.0) | 22 (23.4) | 25 (26.6) | 47 (50.0) | 59 (62.8) | 94 (100.0) | 29  (6.3) | 98 (21.4) | 330 (72.2) | 288 (63.0) | 457 (100.0) |
| **Total [N (%)]** | 211  (49.2) | 112 (26.1) | 106 (24.7) | 255 (59.4) | 429 (100.0) | 57 (21.4) | 76 (28.6) | 133 (50.0) | 149 (56.0) | 266 (100.0) | 87 (8.9) | 278 (28.3) | 616  (62.8) | 611 (62.3) | 981 (100.0) |
| **p-value** | < 0.001 | | | 0.122 |  | 0.679 | | | 0.071 |  | < 0.001 | | | 0.266 |  |

*The p-values for Fisher's tests are linked to the number of patients in categories in Czechia, Hungary, and Poland.*
