# Supplementary material for: Secondary malignancies and survival of FCR‐treated patients with chronic lymphocytic leukemia in Central Europe
Source: Cancer Med. 2022 Oct 7;12(2):1961–71. doi: 10.1002/cam4.5033 (PMC9883578; doi:10.1002/cam4.5033)
Supplement: Supplementary file 6 — Table S6 [file CAM4-12-1961-s004.docx]

Supplementary Table 6. Rate of deceased patients by age groups in CLL patients with secondary malignancies

|  | **Age groups** | | | | | | | | |
| --- | --- | --- | --- | --- | --- | --- | --- | --- | --- |
|  | **18–29** | **30–39** | **40–49** | **50–59** | **60–69** | **70–79** | **80–89** | **≥90** | **Total** |
| **HU [N (%)]** | < 10 (< 2.1) | < 10 (< 2.1) | 13 (2.7) | 58 (11.9) | 123 (25.3) | 204 (42.0) | 76 (15.6) | < 10 (< 2.1) | 486 (100.0) |
| **CZ [N (%)]** | 5 (2.9) | 3 (1.7) | 9 (5.2) | 19 (10.9) | 63 (36.2) | 50 (28.7) | 23 (13.2) | 2 (1.1) | 174 (100.0) |
| **PL [N (%)]** | 15 (0.7) | 21 (1.0) | 46 (2.2) | 268 (12.9) | 575 (27.6) | 735 (35.3) | 408 (19.6) | 17 (0.8) | 2,085 (100.0) |
| **Total [N (%)]** | - | - | 68 (2.5) | 345 (12.6) | 761 (27.7) | 989 (36.0) | 507 (18.5) | - | 2,745 (100.0) |
| In Hungary, the numbers could not be obtained due to privacy regulations if less than 10 patients were reported. | | | | | | | | | |
