# Supplementary material for: Secondary malignancies and survival of FCR‐treated patients with chronic lymphocytic leukemia in Central Europe
Source: Cancer Med. 2022 Oct 7;12(2):1961–71. doi: 10.1002/cam4.5033 (PMC9883578; doi:10.1002/cam4.5033)

**Supplementary Figure 1. Curve to the first secondary malignant neoplasm after CLL diagnosis based on Cox proportional hazard model.**


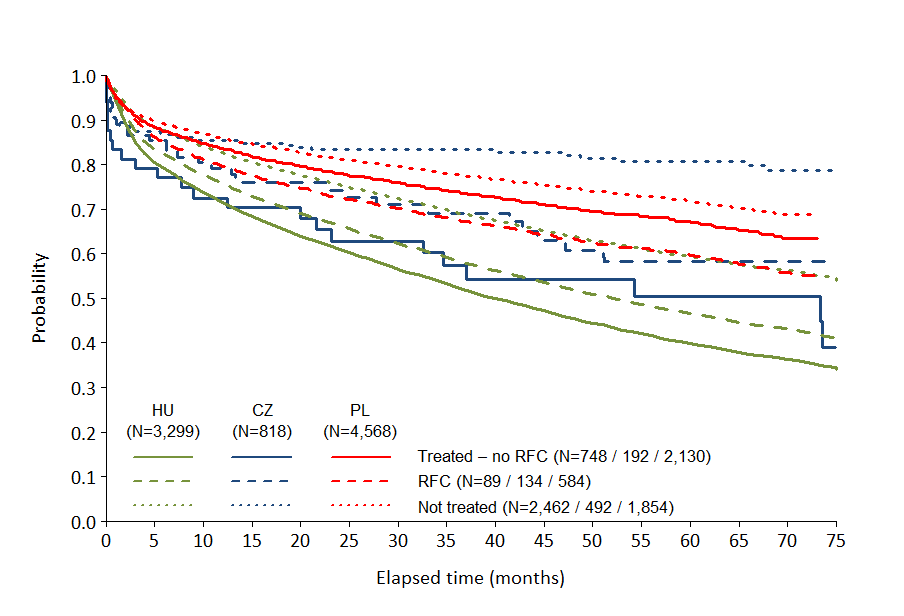

Supplement: Supplementary file 7 — Figure S1 [file CAM4-12-1961-s003.docx]
